# Protocol to analyze chromatin-bound proteins through the cell cycle using Chromoflow flow cytometry

**DOI:** 10.1016/j.xpro.2023.102568

**Published:** 2023-09-18

**Authors:** Dácil Alonso-Gil, Ana Losada

**Affiliations:** 1 Chromosome Dynamics Group, Molecular Oncology Programme, Spanish National Cancer Research Centre (CNIO), Madrid, Spain

**Keywords:** Cell Biology, Flow Cytometry/Mass Cytometry, Molecular Biology

## Abstract

Chromatin-bound proteins have been conventionally measured through subcellular fractionation followed by immunoblotting or by immunofluorescence microscopy. Here, we present Chromoflow, a protocol for the quantitative analyses of protein levels on chromatin in single cells and throughout the cell cycle using flow cytometry. We describe steps for harvesting cells and for nuclear extraction, and a barcoding strategy to multiplex samples from different conditions that reduces antibody staining variability and eliminates the need for normalization.^1^^,^^2^ We then detail procedures for data acquisition and analysis.

For complete details on the use and execution of this protocol, please refer to Alonso-Gil et al. (2023).^3^

## Highlights

• Fast and reproducible analysis of chromatin-bound proteins throughout the cell cycle
• Sample multiplexing avoids antibody staining variability and facilitates comparisons
• Flow cytometry data obtained by Chromoflow are highly versatile and easy to quantify

## Abstract (editor highlights)

Publisher’s note: Undertaking any experimental protocol requires adherence to local institutional guidelines for laboratory safety and ethics.

## Graphical abstract

**Figure:**
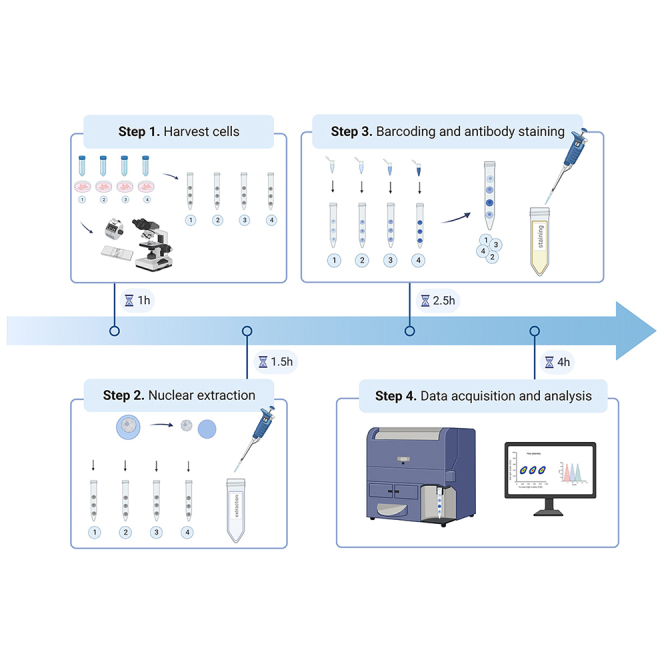

## Teaser

Chromatin-bound proteins have been conventionally measured through subcellular fractionation followed by immunoblotting or by immunofluorescence microscopy. Here, we present Chromoflow, a protocol for the quantitative analyses of protein levels on chromatin in single cells and throughout the cell cycle using flow cytometry. We describe steps for harvesting cells and for nuclear extraction, and a barcoding strategy to multiplex samples from different conditions that reduces antibody staining variability and eliminates the need for normalization. We then detail procedures for data acquisition and analysis.

## Before you begin

### Multicolor panel design

For each experiment samples will require:

1. Barcoding staining. Increasing concentrations of Pacific Blue dye will allow sample multiplexing, which reduces staining variability and facilitates comparisons among conditions. Four different conditions will be barcoded for this particular example: conditions 1, 2, 3 and 4. See Table 1.

**Table 1:** Example of multicolor panel design

Barcoded sample double color staining	Control sample single color staining
STAG1 (rb) + STAG2 (ms) (conditions 1, 2, 3, 4)	STAG1 (rb) + Cy3 anti-rb (dk)
	STAG2 (ms) + AF488 anti-ms (dk)
	Cy3 anti-rb (dk)
	AF488 anti-ms (dk)
MCM3 (rb) + RAD21 (ms) (conditions 1, 2, 3, 4)	MCM3 (rb) + Cy3 anti-rb (dk)
	RAD21 (ms) + AF488 anti-ms (dk)
	Cy3 anti-rb (dk)
	AF488 anti-ms (dk)

Fluorochrome	Emission filter	Excitation laser
Pacific Blue	V_450/50	Violet 405 nm
Alexa Fluor 488	B_525/20	Blue 488 nm
Cy3	YG_586/15	Yellow/Green 561 nm
ToPro3	R_680/30	Red 640 nm
2. Antibody staining. Simultaneous staining with primary antibodies from different host species (e.g., rabbit (rb), mouse (ms), goat) can be performed in the barcoded samples. Secondary antibodies must be from a different host species (e.g., donkey [dk]) and conjugated to fluorophores that are excited by different lasers (in this panel, Alexa Fluor 488 (AF488) and Cy3).
3. Compensation controls are important to correct for spillovers from one fluorophore into secondary channels due to spectral overlap. Thus, single color staining controls must be included in the design.
4. DNA content staining. In this particular panel design, we have chosen ToPro3.

***Note:*** Laser and filters correspond to a BD LSRFortessa cell analyzer. They can be different depending on your panel design. Other fluorochromes can be read with different laser/filters.

***Note:*** Compensation is a basic process in analyses of flow cytometry data. The compensation strategy will depend on the lasers and filters of the cytometer used to detect the fluorophores used in your experiment. Several tutorials about compensation can be found in the internet or as part of the software used for analyses. We recommend that you discuss the compensation strategy of your experiment with an experienced user or person in charge of the Flow Cytometry Facility.

***Note:*** We have omitted a compensation control for Pacific Blue, as the spillover is minor (12% on B_525/20). See Figure S1.

### Cell culture/cell treatment

**Timing: Depending on cell treatment (siRNA: 72 h)**

5. Thaw and plate the cells.
6. Cell treatment. In this particular example, we have knocked down (KD) our proteins of interest (POI) with small interfering RNAs following DharmaFECT transfection protocol (https://horizondiscovery.com/en/transfection-and-ancillary-reagents/products/dharmafect-1-transfection-reagent#resources). Condition 1: mock transfected. Condition 2: STAG1 KD. Condition 3: STAG2 KD. Condition 4: NIPBL KD.

***Note:*** Cell treatment can be either DNA damage induction, transient transfection of a POI or other desired treatments.

### Working solutions to be prepared fresh for each experiment

**Timing: 30 min**

7. Prepare extraction buffer as described under materials and equipment and keep it on ice.

**CRITICAL:** Always prepare a fresh dilution of Igepal CA-630.

8. Prepare staining buffer as described under materials and equipment and keep it at 25°C (room temperature, RT).

***Note:*** Stock solutions of Sodium or Potassium phosphate may precipitate in the cold. Remember to heat and stir until complete dissolution before using it to prepare the staining buffer.

9. Prepare a Pacific Blue *working solution* and *dilutions* as described under materials and equipment when nuclei are extracted (see step 13). Keep them at 25°C (RT).

**CRITICAL:** Make very precise dilutions. It is advisable to use pipets that have been recently calibrated.

## Key resources table

**Table:** 

REAGENT or RESOURCE	SOURCE	IDENTIFIER
**Antibodies**		

Rabbit polyclonal anti-STAG1 Diluted 2 μg/mL	Custom made	Kojic et al.^4^
Mouse monoclonal anti-STAG2 Diluted 2 μg/mL	Santa Cruz Biotechnology	Clone J-12; SC-81852
Mouse monoclonal anti-Rad21 Diluted 2 μg/mL	Sigma-Aldrich	Cat# 05-908
Rabbit polyclonal anti-MCM3 Diluted 1:100	Custom made	Mendez and Stillman^5^
Cy3-Donkey anti-Rabbit Diluted 1:400	Jackson ImmunoResearch	AB_2307443
Alexa Fluor 488-Donkey anti-Mouse Diluted 1:400	Jackson ImmunoResearch	AB_2340846
Pacific Blue	Invitrogen	Cat# P10163
TO-PRO-3 Iodide (642/661)	Invitrogen	Cat# T3605

**Chemicals**		

DharmaFECT 1 Transfection Reagent	Thermo Fisher	Cat# T-2001-03
Opti-MEM I Reduced Serum Medium	Thermo Fisher	Cat# 31985047
Igepal CA-630	Sigma-Aldrich	Cat# i8896
PMSF	Sigma-Aldrich	Cat#10837091001
Formaldehyde solution	Sigma-Aldrich	Cat# F8775
Na_2_HPO_4_	Sigma-Aldrich	Cat# 7558-79-4
KH_2_PO_4_	Sigma-Aldrich	Cat# 7778-77-0
NaCl	Sigma-Aldrich	Cat# 7647-14-5
KCl	Sigma-Aldrich	Cat# 7447-40-7
EDTA	Sigma-Aldrich	Cat# 60-00-4
RNAseA	Roche	Cat# 10109134001

**Experimental models: Cell lines**		

Human: HeLa cells	ATCC	ATCC_CCL2

**Oligonucleotides**		

siRNA targeting sequence: hNIPBL: CUGAUAAACUAGAACGAAA	Dharmacon	N/A
siRNA targeting hSTAG1 (ON-TARGETplus SMARTpool)	Dharmacon	L-010638-01
siRNA targeting hSTAG2 (ON-TARGETplus SMARTpool)	Dharmacon	L-021351-00

**Software and algorithms**		

FlowJo Version v10.4	Tree Star	https://www.flowjo.com
BD FACSDiva Software	BD Biosciences	https://www.bdbiosciences.com/en-us/products/software/instrument-software/bd-facsdiva-software

**Other**		

50 mL tubes	Cultek	Cat# 352098
15 mL tubes	Abdos	Cat# P10402
1.5 mL tubes	Eppendorf	Cat# 0029700
5 mL round bottom polystyrene tube	Falcon	Cat# 352052
Eppendorf 5810R centrifuge, Rotor A 4-81	Eppendorf	N/A
Eppendorf 5415D centrifuge, Rotor F 45-24-11	Eppendorf	N/A
BD LSRFortessa flow cytometer	BD Biosciences	N/A

***Note:*** Narrow, conical bottom of 15-mL centrifuge tubes (e.g., Abdos Cat #P10402) facilitate cell pellet washes.

## Materials and equipment

### Working solutions

**Table:** Extraction buffer

Reagent	Final concentration	Amount
Igepal CA-630 (10%)	0.1%	100 μL
NaCl (5 M)	10 mM	20 μL
MgCl_2_ (1 M)	5 mM	50 μL
PMSF (100 mM)	0.1 mM	10 μL
Phosphate buffer pH 7.4 (0.1 M)	10 mM	1 mL
ddH_2_O	N/A	8.820 mL
**Total**	**N/A**	**10 mL**

***Note:*** Keep on ice and discard after use.

***Note:*** Remember that you will need 1 mL per 2 × 10^6^ cells. Scale as required depending on the number of samples.

***Note:*** Stock phosphate buffer 0.1 M can be prepared either from 1 M Sodium or Potassium phosphate solutions.

**CRITICAL:** Igepal CA-630 concentration in extraction buffer will depend on the cell type. Some cells (e.g., HeLa, MCF10A or HCT116) require 0.1% detergent for successfully extract nuclei while others, such as mouse embryonic stem cells (mESC), are extracted with 0.07%.

**Table:** Staining buffer

Reagent	Final concentration	Amount
Igepal CA-630 (10%)	0.1%	50 μL
Na_2_HPO_4_ (1 M)	6.5 mM	32.5 μL
KH_2_PO_4_ (1 M)	1.5 mM	7.5 μL
KCl (2 M)	2.7 mM	6.75 μL
NaCl (5 M)	137 mM	137 μL
EDTA pH 80.5 M	0.5 mM	5 μL
non-fat milk powder	4%	0.2 g
ddH_2_O	N/A	4761.25 μL
**Total**	**N/A**	**5 mL**

***Note:*** Keep at 25°C (RT) and discard after use.

***Note:*** Scale as required depending on the number of samples.

### Pacific Blue

• Prepare a *working solution* (0.018 μg/μL) and keep it at 25°C (RT):
  ○ 2 μL from a stock solution (5 μg/μL, stored at -80°C) + 553.5 μL DMSO
• Make very precise dilutions in eppendorf tubes and keep them at 25°C (RT):

**Table:** 

Dye level	DMSO	Pacific Blue	Final concentration in DMSO
**L4**	520 μL	200 μL from *working solution*	5.00500 ng/μL
**L3**	300 μL	100 μL from L4	1.25125 ng/μL
**L2**	400 μL	100 μL from L3	0.25025 ng/μL
**L1**	700 μL	100 μL from L2	0.03128 ng/μL

## Step-by-step method details

### Harvest cells

**Timing: 1 h**

This step describes how to prepare samples to extract cell nuclei.

1. Trypsinize adherent cells and harvest in PBS (50-mL centrifuge tube/condition). No trypsin is needed for cells in suspension.
2. Count cells.
3. Centrifuge (5 min at 0.1 RCF, 4°C) and resuspend cells in PBS (1 × 10^6^ cells/mL). Keep your samples on ice.
4. Centrifuge (5 min at 0.1 RCF, 4°C), remove all the supernatant and keep samples on ice.

**Figure 1:**
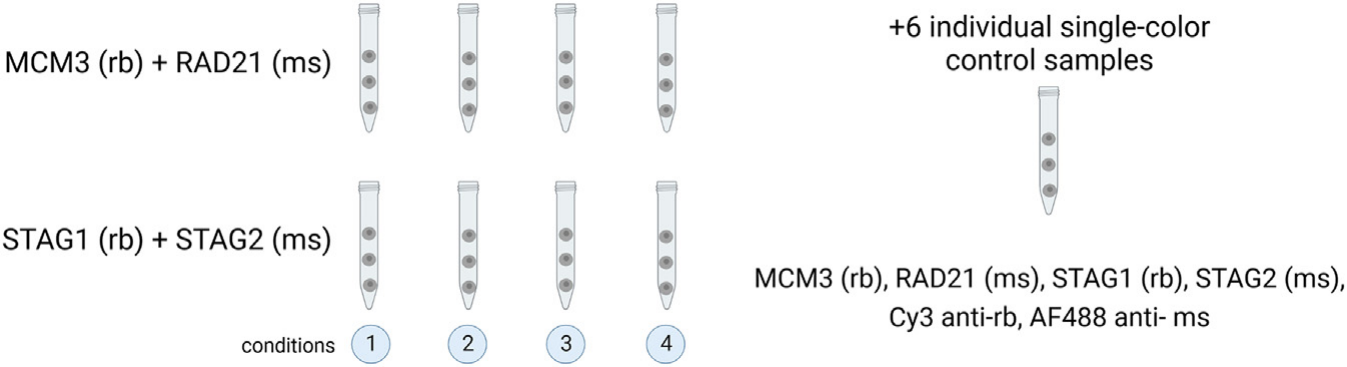
Sample preparation for extraction Each condition and staining tube contain 2 × 10^6^ cells. Total number of cells per condition is 4 × 10^6^. 12 × 10^6^ cells (untreated or mock treated) are split in 6 control tubes. Image created with BioRender.com.

**CRITICAL:** Each tube should contain the same number of cells in order to ensure similar extraction for all conditions.

***Note:*** In this particular example, 2 double stainings of 4 conditions multiplexed in a barcoded sample will require 6 single control colors. Total number of tubes: 14 (2 stainings x 4 conditions = 8, plus 6 single stainings as control samples. See Figure 1.

***Note:*** Cell confluency is very important. Asynchronous cell cycle analyses require no more than 70% confluency. Fully confluent plates will probably enrich the cell cycle profile in G1 phase.

### Nuclear extraction

**Timing: 1.5 h**

This step explains how to treat cells in order to remove the soluble components and leave chromatin-associated proteins.

5. Add 1 mL of extraction buffer to each 2 × 10^6^ cell pellet. Pipet up and down to resuspend cells without making bubbles. See Figure 2.

**Figure 2:**
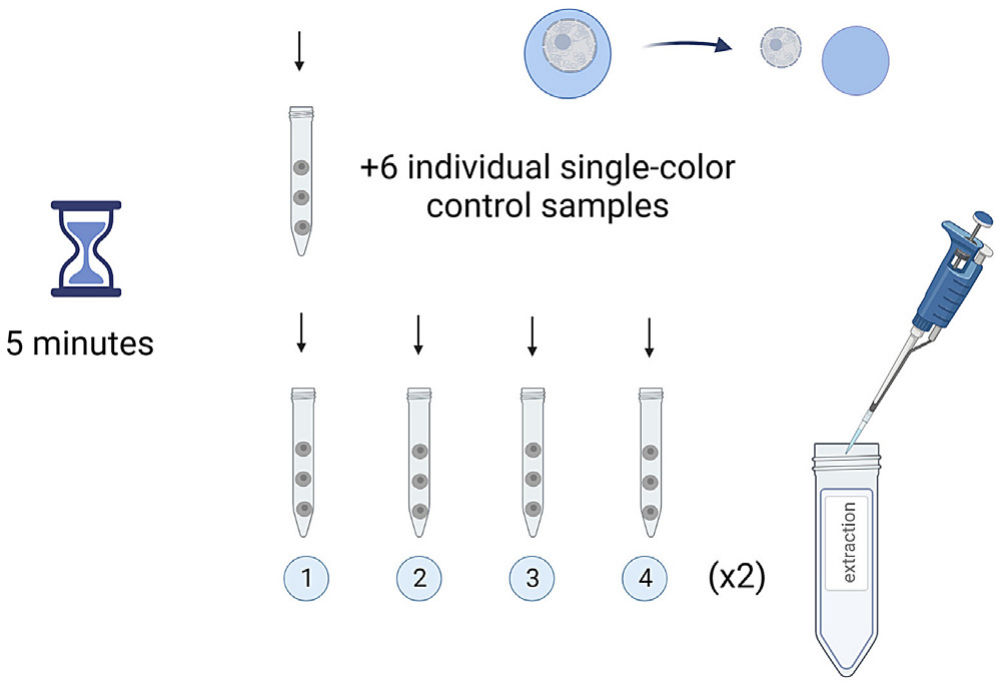
Nuclear extraction Each centrifuge tube containing 2 × 10^6^ cells is treated with 1 mL extraction buffer for 5 min in order to extract soluble nuclear components. Image created with BioRender.com.
6. Place samples back on ice for 5 min.
7. Add 27 μL of 37% formaldehyde solution to the cell suspension (1% final concentration) and incubate for 1 h on ice.
8. Fill the tube with PBS-1% FBS to stop the reaction.
9. Centrifuge (5 min at 0.2 RCF, 4°C) and pour off the supernatant.
10. Resuspend the extracted nuclei in the remaining PBS-1% FBS. See troubleshooting 1**.**
11. Transfer nuclei into a 1.5 mL eppendorf tube.
12. Centrifuge (5 min at 0.8 RCF, 25°C) and strictly remove all supernatant using a micropipettor.

**CRITICAL:** Extraction has to be strictly timed (5 min incubation with extraction buffer). If you have more than 4 tubes (equal to one final barcoded sample), perform several rounds of extraction with no more than 4 tubes per round.

**CRITICAL:** One must be very careful in order not to lose nuclei when transferring them from falcon to eppendorf tubes (steps 10–12).

**CRITICAL:** The proportion of 1 mL of extraction buffer for 2 × 10^6^ cells is very important. Otherwise, extraction may fail.

***Note:*** For control samples (single staining) skip steps 13–19.

***Note:*** Extraction buffer is prepared fresh for each experiment and it must be stored on ice.

***Optional:*** Steps 5–7 are required to analyze chromatin-associated proteins. To analyze total protein content, skip steps 5–7 and instead fix each 2 × 10^6^ cell pellet with 1 mL cold EtOH-70% dropwise. Incubate for 2 h at -20°C and continue to step 8. In case of proteins tightly bound to chromatin, a more stringent extraction can be performed. After step 6 and before fixation, add 20 μL of 5 M NaCl to each tube. This will increase NaCl concentration in 100 mM. Incubate for 5 more minutes on ice and proceed to step 7.

### Barcode the samples

**Timing: 45 min**

This step will dye the nuclei/cells from each condition with a different concentration of Pacific Blue in order to multiplex the different conditions of your experiment in one single tube.

13. Resuspend the fixed pellets in 195 μL PBS (FBS free).
14. Transfer exactly 5 μL of the corresponding dye solution (L1, L2, L3, L4; see **working solutions-**Pacific Blue) into each tube (condition) and mix by pipetting up and down. See troubleshooting 2**.**
15. Incubate samples in the dark for 30 min at 25°C (RT).
16. Fill eppendorf tubes with PBS-1% FBS.
17. Centrifuge (5 min at 0.8 RCF, 25°C) and remove supernatant leaving around 200 μL.
18. Resuspend nuclei/cells in the remaining supernatant and mix samples in one final barcoded tube (L1+L2+L3+L4). From the initial 8 15-mL tubes, you will end up with only 2 eppendorf tubes: one barcoded sample for each staining to be performed in this particular example. See Figure 3.

**Figure 3:**
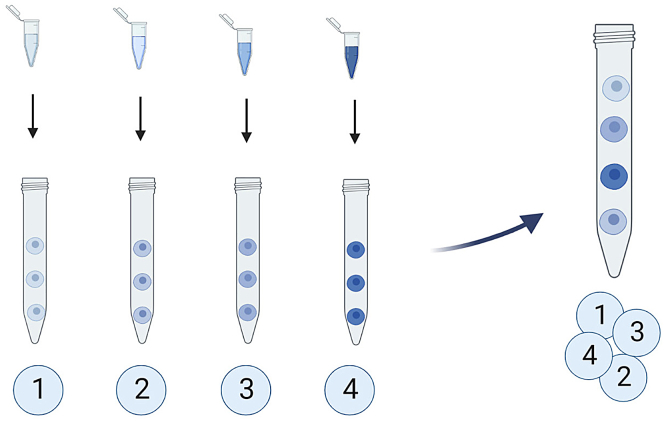
Barcoding strategy L1, L2, L3 and L4 dye conditions 1, 2, 3 and 4 respectively. This barcoding strategy must be followed for each desired barcoding sample. Image created with BioRender.com.
19. Centrifuge (5 min at 0.8 RCF, 25°C) and strictly remove all supernatant with a micropipettor.

**CRITICAL:** FBS will disturb barcoding staining in step 13.

***Note:*** Pacific blue dilutions are prepared fresh for each experiment, and they are kept at 25°C in the dark.

### Antibody staining

**Timing: 1.5 h**

In this step the proteins of interest are recognized by specific antibodies. Table 2 summarizes the antibody staining strategy followed for this particular example.

20. Incubate nuclei/cell pellets for 5 min at 25°C with staining buffer:
  a. Control samples: Add 50 μL of staining buffer.
  b. Barcoded samples: Add 125 μL of staining buffer.
21. Add the same volume of primary antibody staining solutions with the antibody of interest at required concentration taking into account that it will be diluted by half. Incubate for 1 h at 25°C in dark.
  a. Control samples: final volume 100 μL.
  b. Barcoded samples: final volume 250 μL.
22. Fill Eppendorf tube with PBS-1%FBS.
23. Centrifuge (5 min at 0.8 RCF, 25°C) and remove supernatant with a micropipettor.
24. Prepare secondary staining solution at the desired concentration. Incubate for 30 min at 25°C in dark.
  a. Control samples: Add 100 μL of staining solution.
  b. Barcoded samples: Add 250 μL of the staining solution.
25. Fill Eppendorf tube with PBS-1%FBS.
26. Centrifuge (5 min at 0.8 RCF, 25°C) and remove supernatant with a micropipettor.
27. Resuspend each pellet in 0.5 mL PBS (FBS free) + 1 μM ToPro3 + 1 μg/mL Ribonuclease A.
28. Incubate for 30 min at 25°C in dark or store your samples over night at 4°C for analysis next day.

***Note:*** Staining buffer is prepared fresh for each experiment and it is stored at 25°C (RT).

***Note:*** See primary and secondary antibody concentrations for this particular example in key resources table.

***Note:*** Primary and secondary antibodies should have been titrated beforehand to find adequate staining conditions. Recommended dilution for immunofluorescence staining is a good starting point.

***Note:*** Additional washing steps can be performed after primary and secondary antibody staining by repeating steps 22–23 and 25–26, respectively.

**Table 2:** Antibody staining strategy

Barcoded sample double color staining	Control sample single color staining
STAG1 (rb) + STAG2 (ms) Cy3 anti-rb (dk) + AF488 anti-ms (dk) (conditions 1, 2, 3, 4)	STAG1 (rb) + Cy3 anti-rb (dk)
	STAG2 (ms) + AF488 anti-ms (dk)
	No primary // Cy3 anti-rb (dk)
	No primary // AF488 anti-ms (dk)
MCM3 (rb) + RAD21 (ms) Cy3 anti-rb (dk) + AF488 anti-ms (dk) (conditions 1, 2, 3, 4)	MCM3 (rb) + Cy3 anti-rb (dk)
	RAD21 (ms) + AF488 anti-ms (dk)

### Data acquisition

**Timing: Depending on the number of samples (approximately 1.5 h)**

In this step, data are acquired at the flow cytometer and saved for subsequent analyses.

29. Acquire data on BD LSRFortessa or your flow cytometer of choice.
30. Gate a uniform population from Forward Scatter Area (FSC-A) and Side Scatter Area (SSC-A).
31. Gate single cell cycles from DNA content-A (ToPro3-A) vs. DNA content-H (ToPro3-H).
32. Represent single cells in SSC-A vs. Pacific Blue-A to gate the 4 conditions in barcoded samples.
33. Represent DNA content-A (ToPro3-A) vs. your antibody of interest-H (Cy3/AF488-H) and adjust voltages on a linear scale.
34. Record at least 10,000 events for each condition in barcoded samples.
35. Acquire and record 20,000–30,000 events for control samples in order to compensate fluorescence spillover. See step 44 in data analyses**.**
36. Export the Flow Cytometry Standard (FCS) data files.

***Note:*** Voltages for FSC and SCC will depend on the cell line and cell treatment.

***Note:*** Although the fluorophore of secondary antibodies defines the laser to be used, cytometer detector voltages can be different depending on the primary antibody. In this particular example, both *Cy3 anti-rb* and *AF488 anti-ms control* samples have been recorded 2 times, each one with the corresponding voltage of its primary antibody (MCM3 and STAG1 _ Cy3 / RAD21 and STAG2 _ AF488). That makes 8 compensation samples (Figure 5).

***Note:*** Transient transfection of a POI before you begin (e.g., GFP-POI excited by B_525/20 laser) will require extra-gating. After step 33, gate positive cells from *SSC-A vs B_525/20-H* in order to make sure that 10,000 events are recorded for the population of interest.

### Data analyses

**Timing: 2.5 h**

Flow cytometry data can be analyzed in multiple ways depending on the question. Quantitative analyses comparing the amounts of chromatin-bound proteins between different conditions (control and KD) and at specific cell cycle phases can be easily performed. For the example described here, we have used FlowJo software for analysis. However, alternative software such as FCS express or ModFit can be considered as well.

37. Drag and drop the FSC files and repeat gating strategy (steps 30–32).
38. Export single FSC files for each population of barcoded samples. Make sure exports contain the same number of events (10,000 cells). See Figure 4.

**Figure 4:**
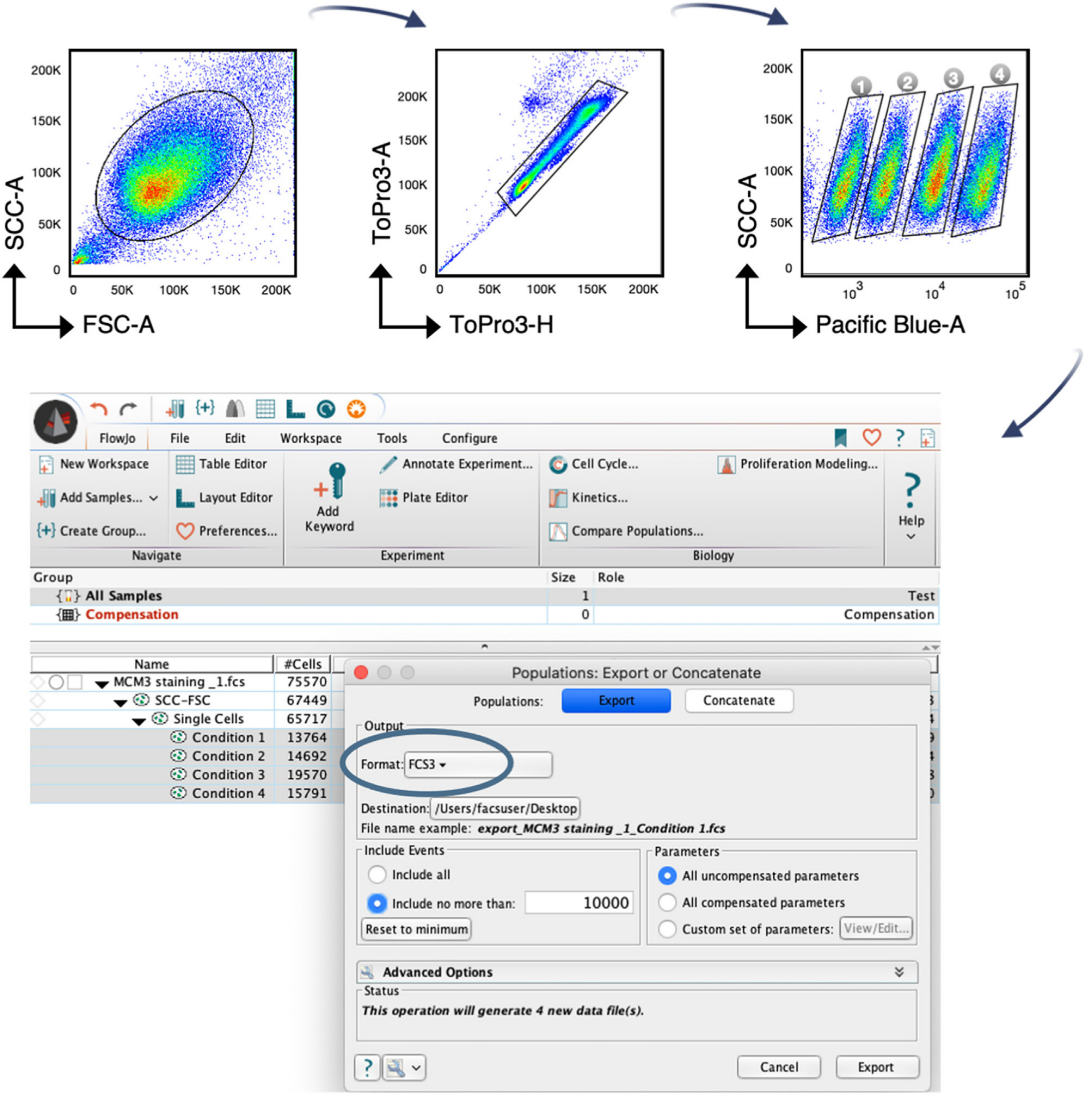
Gating strategy for data analysis Gating strategy for uniform population (top left), single cell cycles (top middle) and different conditions in your experiment (top right) is required to export single FSC3 or CSV-scale values files (bottom).
39. Export single cell cycles of control samples (20,000–30,000 cells each) with the same gating strategy as steps 30–31.
40. Create a new workspace with *staining groups* in order to apply compensation parameters.
41. Drag and drop barcoded files into their corresponding *staining group.*
42. Drag and drop control samples files into the compensation folder in FlowJo software.
43. Apply sample quality (green/blue: good quality; pink: poor quality in FlowJo).
44. Use specific color compensation for each corresponding *staining group.* See Figure 5.

**Figure 5:**
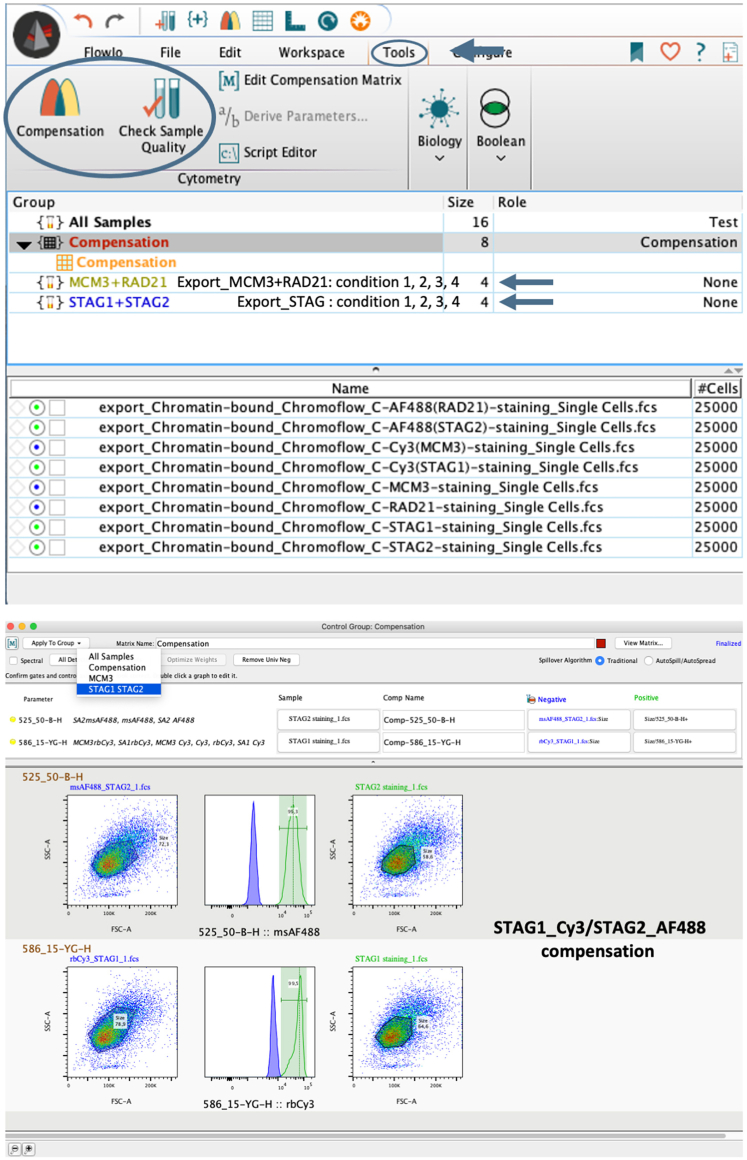
Data compensation Specific compensation parameters (given by control samples) are applied to each staining group containing barcoded samples.
45. Create a layout.
  a. Drag and drop your samples.
  b. Represent compensated-antibody-H vs. DNA content-A to observe the cell cycle profile of your protein of interest.
  c. Compare conditions among the same barcoded sample. See Figures 6A and 7.

**Figure 6:**
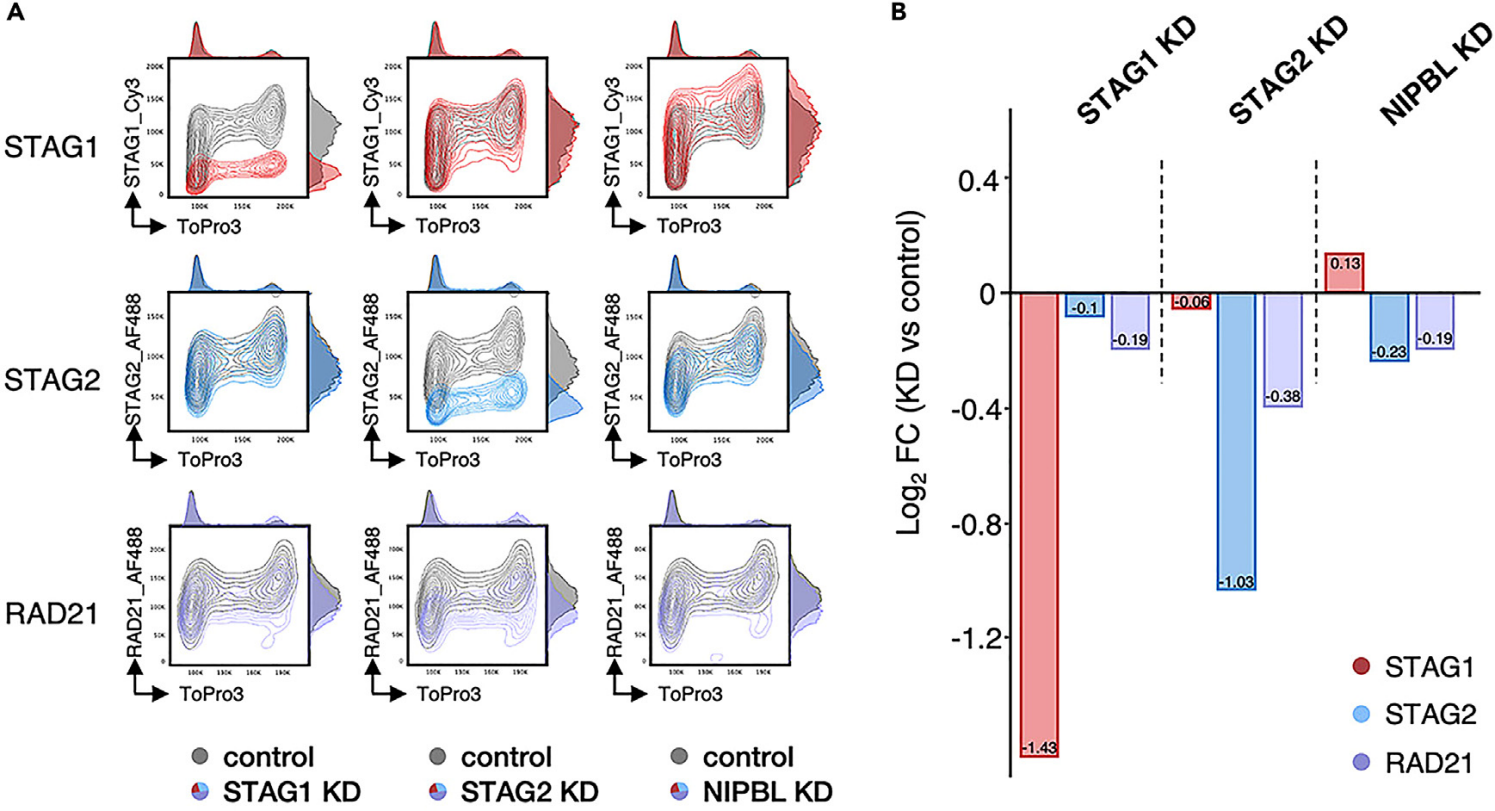
Data representation (A) Contour plots of exported FCS3 files. Compensated antibody of interest-H vs. DNA content-A is represented with adjacent histograms in a linear scale. Contour plots for STAG1, STAG2 and RAD21 in control (gray) and STAG1 KD, STAG2 KD or NIPBL KD conditions (colored) are overlapped for comparison. Data reanalyzed from Alonso-Gil et al. (2023). (B) Same data as A. Quantified median values from the exported CSV-scale values files were used to study log2 fold change (FC) enrichment of STAG1, STAG2 and RAD21 in all conditions.

**Figure 7:**
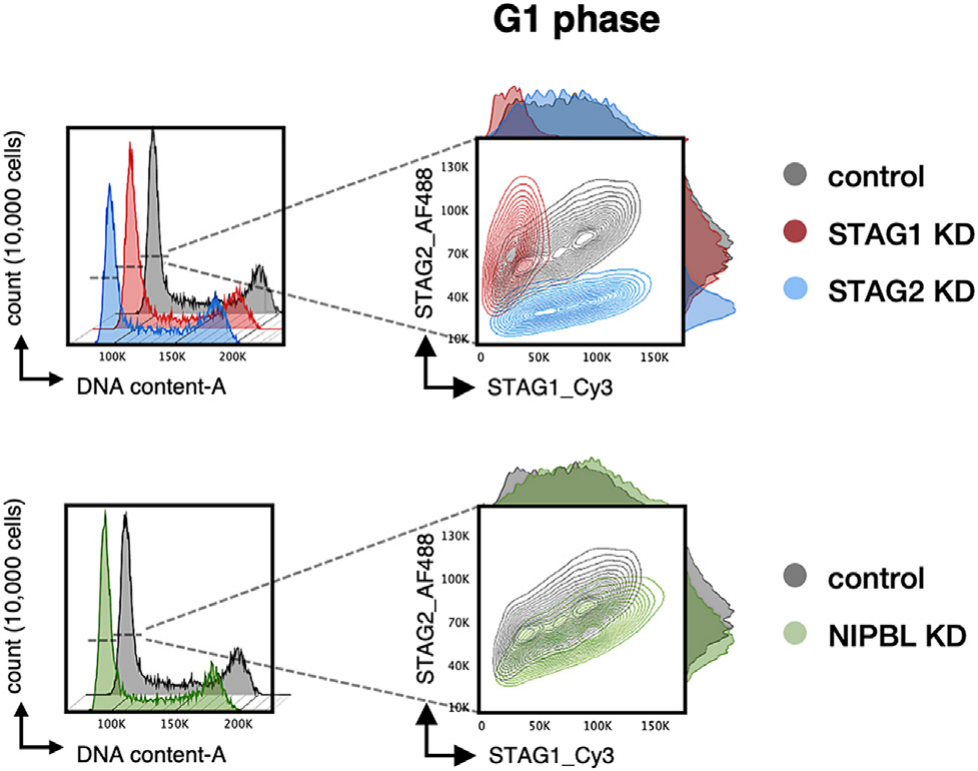
Versatile analyses Among other analyses, the behavior of your POI at specific moments of the cell cycle can be addressed. In this particular example, cell cycle representation as a DNA content-A histogram (left) allows to manually gate cells in G1 phase. Next, the amounts of STAG1 and STAG2 in these G1 cells can be plotted (right) and compared between control (gray) and KD conditions (colored).

***Note:*** In steps 38–39, files can be exported in other formats than *FCS3* (Figure 4), such as *CSV-scale values* in order to allow for data quantification. See Figure 6B.

## Expected outcomes

**Figure 8:**
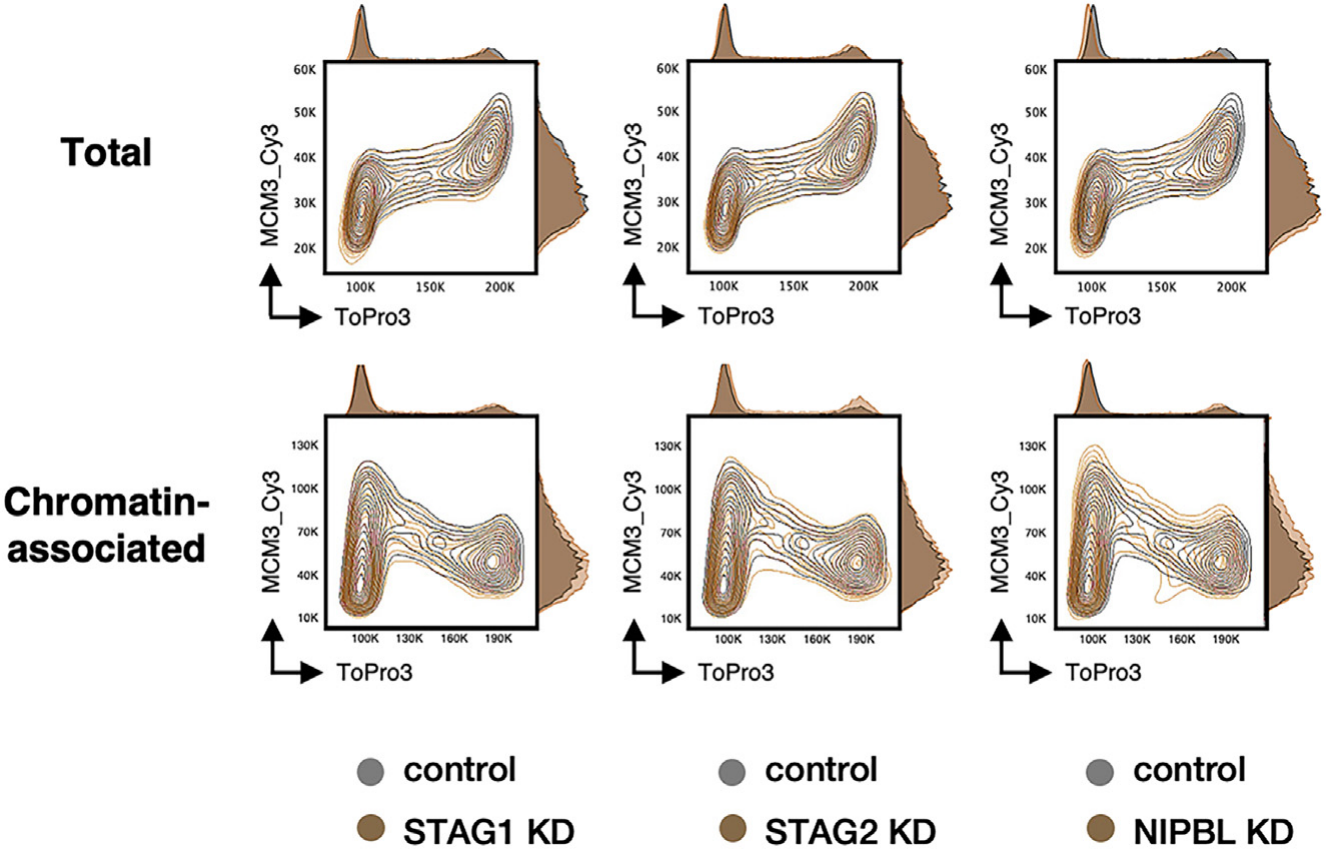
Cell cycle profile of MCM3 Contour plots of exported *FCS3* files. Compensated MCM3 antibody staining-H vs. DNA content-A is represented with adjacent histograms in a linear scale for total and chromatin association protocols (see optional step from nuclear extraction in step-by-step method details). Contour plots for MCM3 in control (gray) and STAG1 KD, STAG2 KD or NIPBL KD conditions (colored) are overlapped.

Staining and cell cycle analysis of MCM3 discloses successful extraction of soluble proteins. Although MCM3 profile shows a continuous increase from G1 to G2 when total proteins are analyzed (Figure 8 top), its chromatin-bound profile reveals that the protein loaded during G1 phase is progressively released by the passage of the replication fork during S phase (Figure 8 bottom).

## Limitations

This method relies on the availability of high-quality antibodies that specifically recognize your POI and that work for flow cytometry.

Unlike immunofluorescence microscopy, Chromoflow does not provide information of the localization of the POI within the nuclear space. Treatments that affect protein distribution without changing total chromatin-bound levels will not show any difference in this assay.

The binding mode of your POI to DNA will dictate its sensitivity to the extraction method. Optimal extraction may require increasing the amount of salt in the extraction buffer, as suggested above.

## Troubleshooting

### Problem 1

Related to steps 10–12, extracted nuclei may be progressively lost during the following centrifugation steps.

### Potential solution

Resuspending samples in PBS-1%FBS will help pellet the nuclei before transfer from 15-mL tubes to eppendorf tubes or during the washing steps.

### Problem 2

For barcoding (steps 13 and 14), volumes must be exact. Otherwise, the four different conditions in the barcoded sample may not be properly separated upon data acquisition and data analyses.

### Potential solution

• Ensure that all supernatant is removed in step 12 and fixed pellets are resuspended in 195 μL PBS (FBS free). Then, add exactly 5 μL of the dye dilution to the suspension.
• Calibrate/adjust micropipettes.

## Resource availability

### Lead contact

Further information and requests for resources and reagents should be directed to the lead contact, Ana Losada (alosada@cnio.es).

### Materials availability

No new materials are generated in this protocol.

## Data Availability

• Data supporting the current study has been published in https://doi.org/10.1038/s41467-023-36900-7. • This paper does not report original code.
