# Supplementary material for: Protocol to analyze chromatin-bound proteins through the cell cycle using Chromoflow flow cytometry
Source: STAR Protoc. 2023 Sep 18;4(4):102568. doi: 10.1016/j.xpro.2023.102568 (PMC10510066; doi:10.1016/j.xpro.2023.102568)
Supplement: Document S1. Figure S1 [file mmc1.pdf]

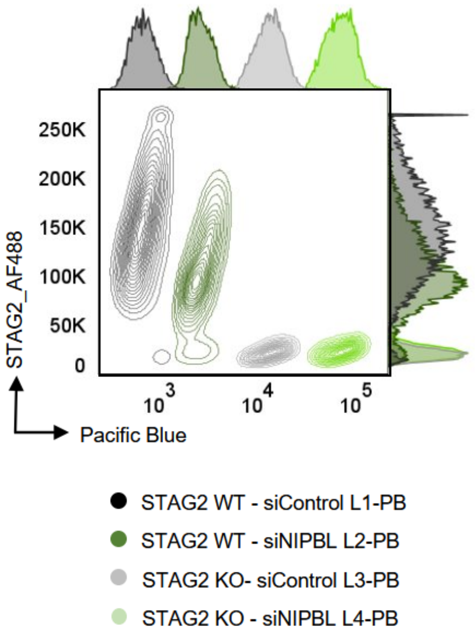

Supplementary Figure 1. Pacific Blue does not spill over AF488 staining, Related to Table 1 in Multipanel design, “Before begin”.

STAG2 AF488 staining in cells with (WT) and without (KO) STAG2 showing that high PB concentrations (L3 and L4) do not spill onto B_525/20, supporting the omission of compensation for PB in the example of Table 1.
